# Disgust sensitivity and psychopathic behavior: A narrative review

**DOI:** 10.1515/tnsci-2022-0358

**Published:** 2024-12-11

**Authors:** German Torres, Amina A. Sheikh, Beatrice G. Carpo, Riya A. Sood, Mervat Mourad, Joerg R. Leheste

**Affiliations:** Department of Biomedical Sciences, New York Institute of Technology College of Osteopathic Medicine (NYITCOM), Old Westbury, New York, 11568, United States of America; Department of Clinical Specialties, New York Institute of Technology College of Osteopathic Medicine (NYITCOM), Old Westbury, New York, 11568, United States of America

**Keywords:** olfactory processing, criminality, disgust sensitivity, psychopathy, parasite avoidance, dysbiosis, emotional expression, sensory integration, psychiatric disorders, antisocial behavior, amygdala, disgust, prefrontal cortex, insula, gastrointestinal tract, olfaction

## Abstract

Humans live under constant threat from pathogenic microorganisms and minimizing such threat has been a major evolutionary selective force in shaping human behavior and health. A particular adaptive mechanism against the harm caused by parasites and their infectiousness is disgust sensitivity, which has evolved to detect and avoid poisonous foods as well as bodily secretions harboring virulent microorganisms. This ubiquitous and reflexive behavior requires the integration of several internal and external sensory signals between the brain, the autonomic nervous system (ANS), and the gastrointestinal tract. Although the emotional expression of disgust is experienced by almost all individuals, the neural mechanisms of sensory signals underlying disgust sensitivity may differ in certain psychiatric conditions. Psychopathy, for instance, is a personality disorder in which disgust sensitivity to contagious bodily secretions is apparently absent or downregulated from its atypical personality temperament. In this review, we provide convergent behavioral, anatomical, and cellular evidence to suggest that a fractured experience of disgust sensitivity might be an additional feature of psychopathic behavior. First, we discuss the neural networks of certain brain regions mediating the emotional states of disgust and then discuss the intersection of the ANS and gastrointestinal tract in the processing of disgust and its relevance to aberrant antisocial behavior. Together, this work highlights the interconnections between the brain and the bilateral body plan as an integrated cell network that is relevant for understanding common principles underlying function and dysfunction of disgust levels in psychiatric domains.

## 1 Introduction

Humans have faced starvation, predation, and infection throughout their unique evolutionary history. Infection, in particular, has been a ubiquitous variable in human evolution, as pathogens and parasites can influence disease transmission and virulence. Thus, avoidance adaptations to withstand or limit parasitic burdens must have been under strong selection pressure since the appearance of anatomically modern humans over 400,000 years ago. Indeed, the development of emotional behaviors such as disgust has yielded the appropriate mechanisms for mitigating the transmission of infectious diseases through cognitive and autonomic nervous system (ANS) strategies. It would thus seem reasonable to assume that disgust as a disease-avoidance mechanism has ancestrally evolved, innately conserved, and genomically propagated across human populations because it has a strong survivorship bias. Here, we consider the implications of disgust sensitivity in criminal behavior, as downregulation of this adaptive system is often observed in personality disorders, namely psychopathy. More broadly, we hypothesize that deficits in disgust sensitivity may be a physiological marker of this particular personality disorder. To accomplish this consideration, a systematic review of the literature was performed based on PubMed databases with articles published between 2010 and 2024. We adopted criteria from the literature to categorize psychopathic behavior as described in both the DSM-5 and the triarchic model of psychopathy. Also, the psychopath label is only used to describe those individuals who have come to the attention of criminal law authorities and/or psychiatric institutions.

## 2 Background

**Figure 1:**
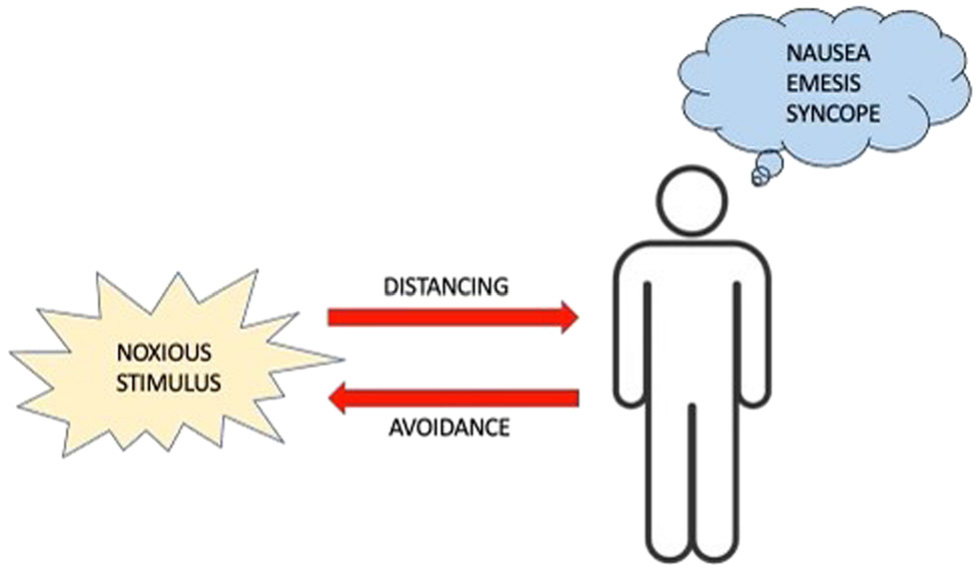
A schematic diagram depicting the function of disgust as a disease-avoidance mechanism. The perception and sensation of disgusting or repulsive biological material can alert an individual that a specific type of food or a particular bodily secretion is harmful and should not be consumed or approached (noxious stimulus). Distancing oneself from and avoiding contact with pathogens and parasites are cognitive strategies evolved to recognize the threat they pose in (unfamiliar) social interactions [3]. Disgust also incorporates ANS strategies to avoid or prevent exposure to microorganisms and toxic constituents. Nausea, emesis, and syncope represent a diverse array of defensive behaviors that can reflexively be induced when threatening viruses, bacteria, or poisonous chemicals enter the bilateral body plan either *via* the enteral (i.e., the gastrointestinal tract) or parenteral route (i.e., skin, mouth, and blood). Differences in the intensity or degree to which disgust is experienced appear to be common variables in certain psychopathologies. Thus, disgust sensitivity may have a broader range of biological functions than just alerting the individual to harmful threats.

According to current thinking, disgust sensitivity protects the individual from pathogen infection and ultimately shapes communal hygiene behavior [1,2]. Extensive theoretical analyses of the variables that shape the evolution of disgust sensitivity have been reported in the last two decades [3–5]. By and large, conventional theory states that body odors, excreta, saliva, sexual biofluids, blood dripping, and wound secretions elicit disgust responses in most individuals in the form of personal distancing and contact avoidance (cognitive strategies) and nausea, emesis, and syncope (ANS strategies). Note that the elicitors of disgust behavior are natural sources of infection, as they often harbor soil-transmitted helminths (e.g., hookworms), ectoparasites (e.g., scabies and pediculosis), pathogenic viruses (e.g., Zika), and bacteria (e.g., Treponema pallidum). Of course, there are other aversive stimuli that provoke disgust in humans: spoiled food, for instance, as well as volatile organic compounds associated with cadaveric decomposition (Figure 1).

**Figure 2:**
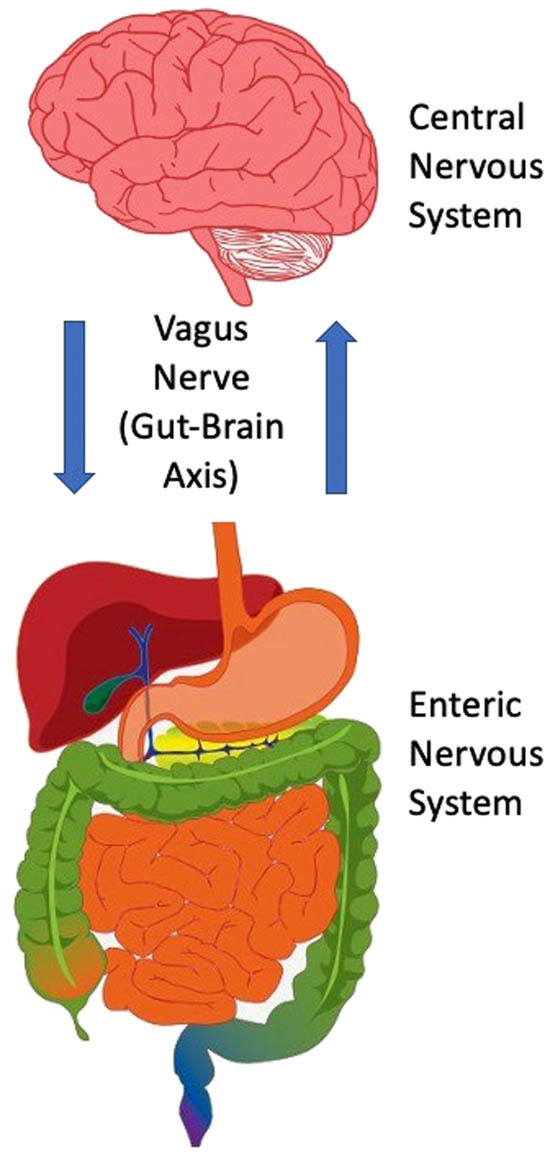
A schematic diagram depicting the interaction of the brain and gastrointestinal tract in the processing and signaling outcomes of disgust. This bidirectional communication takes place *via* the vagus nerve, which has acquired a special significance as a viable target for treating various neurological and psychiatric disorders (e.g., refractory epilepsy and chronic treatment-resistant depression, respectively). The vagus nerve converts interoceptive information into electrical signals that stream up to the visceral hindbrain (medulla) and then subsequently to neural circuits of the cognitive cortices (prefrontal, orbitofrontal, insula), emotional limbic systems (amygdala), and neuroendocrine-secreting sites (hypothalamus). On the basis of this circuitous pathway, it is clear that the emotional expression of disgust recruits a broad cell network that primes affective-behavioral states to limit harm from opportunistic pathogens. The fact that the brain and gastrointestinal tract (including the enteric nervous system) are entangled to generate levels of disgust has important implications for our ability to understand personality disorders such as psychopathy.

As most virulent microorganisms involved in disease threats are too small to detect (ranging from about 20 nanometers in diameter for viruses to 200 nanometers in diameter for bacteria), humans rely on indirect cues that signal the risk of infection [6,7]. Thus, the smell of malodorous indoles from fecal material or the sight of oral and body biofluids, skin and soft tissue lesions, secretions from open wounds, and bleeding from genito-anal areas are salient cues used to detect and assess threats of infection. The fact that disgust has a strong emotional component with cognitive and ANS involvement suggests that different cell signaling pathways, working simultaneously as a network and exhibiting crosstalk, guide the expression of disgust behavior [5,8,9]. Indeed, distinct emotions such as disgust- and fear-related events appear to involve the amygdala, a subcortical brain structure, whose role in processing emotions and motivational behaviors is already well known [10]. Studies using functional magnetic resonance imaging (MRI) also show that the orbitofrontal cortex and the anterior insular cortex are parts of the brain that help process feelings of disgust or revulsion [11]. In this context, the anterior insular cortex appears to be associated with the perception underlying heightened alertness and emotional awareness of aversive conditions [12–14], as well as the interoceptive attention of gastrointestinal tract activity [15]. The crosstalk between brain neurons and the gastrointestinal tract (i.e., the brain-gut axis) provides a point of reference for deciphering the cell circuits processing, encoding, and generating self-awareness of smells and sights of noxious biological material (Figure 2).

The sight of highly contagious bodily biofluids also elicits unique responses from the ANS in the form of nausea, emesis (i.e., vomiting), and, in rare cases, syncopal episodes [16]. It is generally assumed that nausea *facilitates* the avoidance of toxic constituents, whereas emesis or regurgitation *clears* untoward contents from the stomach [17]. Regardless of their specific preventative functions, both nausea and emesis are ancient and ubiquitous survival reflexes involving chemosensitive receptors, efferent and afferent pathways of the gastrointestinal tract, and sensory neurons of the central nervous system (CNS) and the ANS [18]. It should be noted that there is no isolated emetic center but rather clusters or nesting of loosely organized neurons that sculpt the vestibular system, the area postrema, and its adjacent nucleus: the nucleus tractus solitarius; the ventral medulla in the hindbrain; and the hypothalamus [19–22]. However, it is generally accepted that afferent fibers from the gastrointestinal tract and the vagus nerve (cranial nerve X) are arguably the most important pathways coordinating the sequence of behaviors during the self-awareness of unseen pathogens and the forceful expulsion of stomach content [23,24].

## 3 Individual differences in disgust sensitivity

There are individual differences in the intensity or degree to which disgust is experienced. These differences are thought to be related to the emotionality and nervous temperament of a person [25]. As emotionality and nervous temperament are personality traits with a complex genetic architecture coupled with unique environmental experiences, disgust as a disease-avoidance behavior should not be viewed as a fixed trait but rather as a particular trait that is phenotypically malleable and expressed within the statistical mean range for behavioral dispositions. That is, the intensity of disgust can vary from one encounter to the next, depending on internal (e.g., molecular processes underlying individual development) and external (e.g., personal contextual influences) factors. For these and other reasons, sensitivity or proneness to disgust appears to be manifested through distinct causal dimensions encapsulating the totality of human life, from the prenatal period onwards to the adulthood phase and beyond.

It is generally agreed that nervous temperament, social attachment, empathy, frustration, and aggression are behaviors developed in infancy [26,27]. As in disgust or revulsion, nearly every aspect of human behavior is mediated by its unique inherited genetic code, neural activity of networks, physiological signals, and structural connectivity, reinforced along the way by a unique life-exposure trajectory. Thus, the biology of early-life as well as adult-life experience must be considered when attempting to further understand the causal signaling pathways underlying behavior [28]. Given well-established links between nervous temperament and onset of a particular behavior, questions about normative and atypical behaviors are of special significance because of the possibility that perinatal brain injury, low-grade inflammation of the gastrointestinal tract, or protracted maturation of reflective processing and cognitive control might be associated with mental health outcomes. Indeed, heightened or downregulated behavior as a consequence of a fractured biological or cognitive system might be a feature of psychopathology, namely personality disorders such as psychopathy.

## 4 Psychiatric disorders: Psychopathy

**Figure 3:**
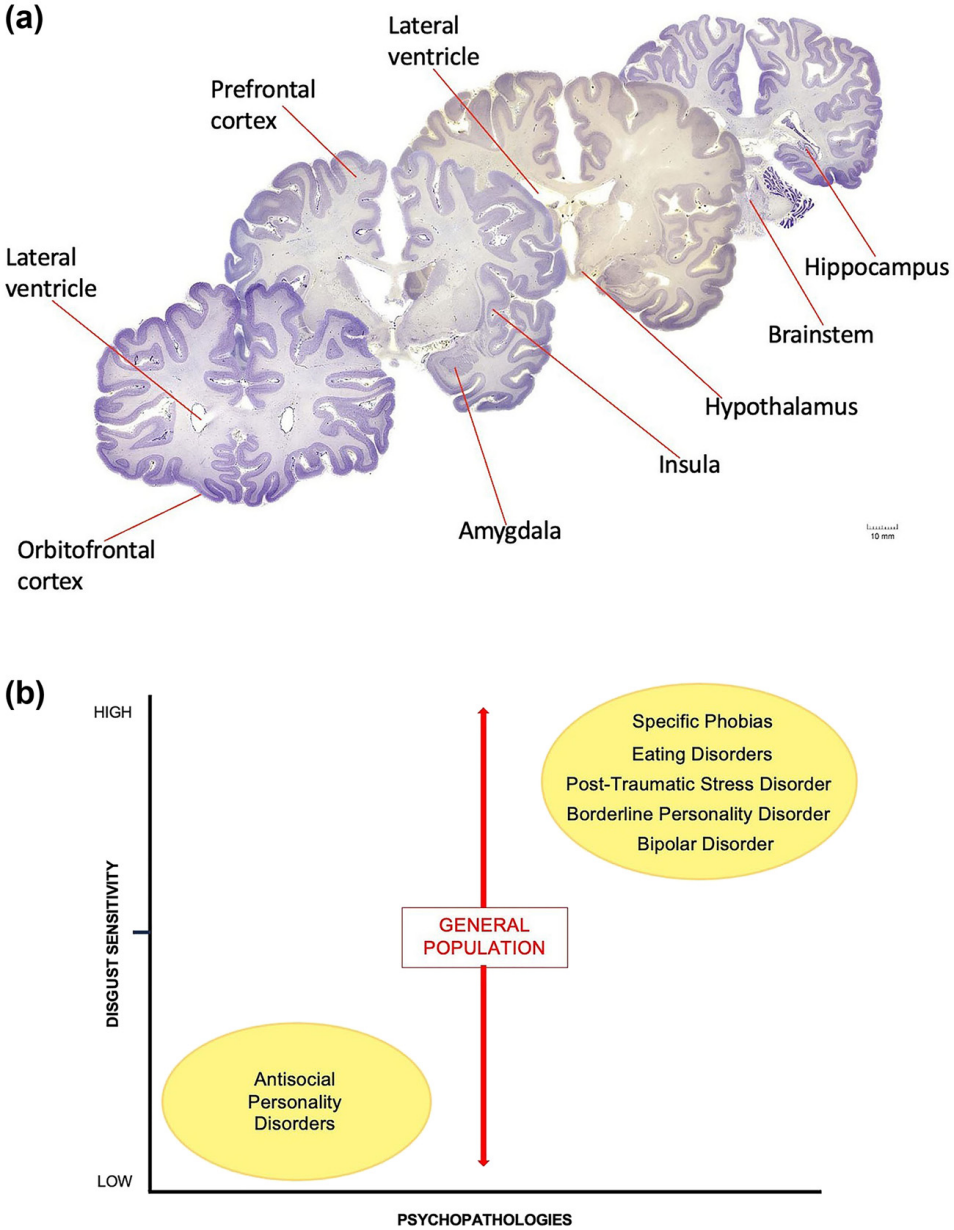
(a) Coronal sections of the human brain. (b) Schematic graph illustrates differences in the intensity or degree to which disgust is experienced in several psychiatric disorders relative to the general population. Subjects diagnosed with psychopathy display a number of aberrant behaviors that are potentially linked to certain anatomical regions of the brain (a). For example, neurons of the prefrontal, orbitofrontal, and insular cortices, neurons of the amygdaloid complex, hypothalamic neurons, and neurons of the medullary reticular formation (within the brain stem) establish synaptic connections both within and with other nerve cells to form distinct circuits underlying normative and perhaps psychopathic behavior. It is now thought that damage to the chemical synapse or circuit formation during development could lay the foundation for personality disorders. The degree of shared brain regions between disgust and psychopathy suggests that the same discrete biology could underlie both personality traits. We hypothesize that disgust sensitivity is apparently absent or downregulated in subjects diagnosed with psychopathy. In sharp contrast, however, increased or heightened sensitivity levels of disgust are frequently diagnosed in other personality disorders (b). While social and cultural factors certainly contribute to these differences, a broad range of physiopathogenic mechanisms within the behavioral spectrum of each syndrome may also be involved. Although impulsivity, hostility, obsessive–compulsive characteristics, affect dysregulation, personality dissociation, and other obtrusive temperamental traits might be shared by both psychopaths and patients with different psychiatric disorders, levels of disgust sensitivity (*X*-axis on the schematic graph) appear to considerably differ among certain psychopathologies (*Y*-axis on the schematic graph). Human brain sections were obtained from the Michigan State University, Brain Biodiversity Bank; supported by the National Science Foundation.

The Diagnostic and Statistical Manual of Mental Disorders (Fifth Edition, DSM-5) classifies psychopathy as a personality disorder characterized by callous aggression and a lack of affective interpersonal values for others. These dispositional traits are also cataloged in the triarchic model of psychopathy: boldness, meanness, and disinhibition [29]. In brief, boldness refers to diminished negative affect; meanness refers to limited attachment to others; and disinhibition refers to poor behavioral constraint. Although psychopathy is often characterized in terms of behavioral metrics and forensic analysis, the genetic and neurobiological underpinnings of the disorder are unclear [30]. Nevertheless, aberrant brain networks and neural developmental defects are thought to contribute to the inability to integrate affective-interpersonal values for others, a hallmark of a psychopathic personality [31–33]. Neuroimaging studies (e.g., high-resolution anatomic MRI) show impairments of the amygdala, orbitofrontal cortex, and paralimbic structures (e.g., anterior insular cortex) in the form of gray matter reduction and synaptic connectivity in subjects diagnosed with psychopathy [34–37]. Interestingly, these are the same brain regions that function synergistically to translate the emotions of disgust into particular outcomes, namely the avoidance of infectious diseases (Figure 3a). Environmental risk factors such as social isolation and childhood trauma are considered to be among the causes of psychopathy [38]. Psychopathy may have its origins *in utero*, with obstetric risk factors including complications such as low birth weight and an emergency cesarean section [39]. Along the same lines, fetal insults such as maternal stress and viral or bacterial infection during pregnancy have also been proposed as risk factors [40]. In regard to genetics, no candidate genes have been put forward to enable the identification of molecular signals or chromosomal sites driving and sustaining psychopathic behavior.

## 5 Psychopathy and disgust sensitivity

Psychopathy, as previously mentioned, is characterized by callous aggression and a lack of affective-interpersonal values for others; behavioral disorders that are potentially based on aberrant brain structure and flawed neural connectivity [41–44]. In addition to the above disorders, there are other features of psychopathy that are closely related to disgust. For instance, most psychopaths often participate in repeated violent criminality that puts them directly in contact with pathogens and parasites. More specifically, during their interpersonal crimes, contagious substances such as body secretions (e.g., blood, saliva, urine, and/or fecal material) from their victims enter psychopaths not only *via* oral ingestion (see below) but also through the eyes, nose, skin, or sexual organs. As noted, bodily biofluids may be laden with unseen microbes and are therefore potential hosts for infectious diseases. Thus, the basic emotion of disgust that serves as a disease-avoidance mechanism appears to be lacking in psychopathy. As disgust sensitivity is an adaptive mechanism that evolved in many vertebrate organisms for the purpose of mitigating the risk of infection, the absence or downregulation of this ancestral behavior requires further evaluation. In this context, disgust sensitivity has so far been outside the mainstream of criminal behaviors, especially those dealing with diminished ability to integrate affective-interpersonal values for others.

A proportion of psychopaths also frequently participate in paraphilic behaviors that increase their contact with microbial threats. For example, necrophilia and cannibalism are two deviant behaviors that carry with them significant risks of infectious diseases. Cadaveric decomposition involves the release of organic compounds such as acetic acids, phenols, and indoles derived from the activity of epinecrotic bacteria [45]. These compounds are by-products of cadaveric decay and are primarily responsible for the noxious odors associated with death. Cadavers also harbor potentially carcinogenic biogenic amines such as cadaverine and putrescine that carry with them enteric viruses such as SARS-CoV-2 [46]. This pathogenic virus, as well as other bacteriophages, contaminates crime-scene locations, further increasing the probability of infection for psychopaths engaged in necrophilic acts. Postmortem bodies also harbor arthropods and other cadaver-associated organisms (e.g., mites, maggots) that may serve as hosts or vectors for pathogens and parasites. Taken together, these observations suggest that certain psychopaths have a general deficit of disgust, leading them to carry out a variety of paraphilic behaviors (e.g., necrophilia) that put them at risk of infectious and transmissible diseases.

Distancing and avoiding contact with body secretions are cognitive strategies often used to mitigate the risk of contracting pathogenic infections [47]. Ingesting contaminated biological material would clearly have a deleterious impact on health and would therefore be evolutionary advantageous not to consume potentially infected conspecifics. However, cannibalism is a widespread behavior in animal taxa, from invertebrates to mammals, including humans [48–50]. Consequently, cannibalism cannot be regarded as a deviant behavior unless performed in the context of pathological criminal activity. While cannibalism has clearly adaptive foraging benefits in the natural world (e.g., during periods of starvation or famine), the prevailing view is that consuming conspecifics increases considerably the risk of contracting pathogenic infections [51]. For instance, humans and other mammals carry with them infectious proteins such as prions (PrP^Sc^), which are responsible for transmissible spongiform encephalopathies as well as Creutzfeldt–Jacob, Kuru, and Gerstmann–Straussler neurodegenerative diseases [52]. Collectively referred to as prion diseases, prions are misfolded cell-surface proteins that can spread within and between cells and can transmit disease between conspecifics, causing nerve cell loss, reactive gliosis, aggregated insoluble fibrils, and inflammation of the brain parenchyma [53,54]. Thus, incidents of exposure to prions can occur in subjects that impulsively engage in pathological criminal cannibalism. An intriguing possible outcome of this observation is that some psychopaths might have diminished levels of revulsion, which goes against the predicted theory of disgust, which states that reducing contact with pathogens and parasites has been under strong selection pressure and hence will be ubiquitous in all humans. Here, certain individuals low in disgust tendencies tend to react less frequently and less intensely with disgust to different noxious stimuli. Thus, downregulation of disgust sensitivity may therefore be an additional feature of psychopathy that requires careful evaluation. It should be noted that although there has been some progress in understanding the concept of psychopathy [55], our knowledge about the brain mechanisms underlying psychopathic behavior is still limited. This is mainly because the human brain is particularly complex in its cellular architecture and signaling interactions. Additionally, the etiology of psychopathy is difficult to assess due to its various clinical manifestations, including comorbid traits with other psychiatric disorders, and the fact that many environmental variables may lead to behavioral dysfunction.

## 6 General discussion

At first glance, the proposed hypotheses discussed in this narrative review may seem to have little in common. Yet on close scrutiny, it becomes apparent that there is an intriguing degree of overlapping features between disgust sensitivity and psychopathy. Although psychopathy in general clearly involves biological abnormalities and environmental degradations that give rise to an atypical personality temperament [56,57], the boundaries of psychopathy might also extend to functions and dysfunctions of an ancestral innate behavior, namely disgust sensitivity.

Psychopathology has long been a subject of interest to psychiatrists, psychologists, neuroscientists, police enforcement, criminal law, and criminal justice. In the case of psychopathy, the prevailing view is that this is a dangerous personality disorder with little chance of recovery, no reliable pharmacological or therapeutic intervention available, and demands from the general public for maximum punishment, including the death penalty for offenders [58]. As psychopathy is not well understood at the molecular or cellular level for diagnostic and informative correlates, most studies of violent offenders have been aimed at identifying common signs and symptoms of childhood predictors to establish a pathological background for risk recognition, disorder management, and perhaps early prevention [59–61]. In clinical settings, certain psychopathic personality signs can be detected in childhood and adolescence, including a lack of empathy and shallow emotion, as well as proactive aggression (i.e., antisocial spectrum disorders) [62,63]. In experimental settings, low resting heart rate and nocturnal enuresis have been linked to neurodevelopmental immaturity and increased risk for psychopathology [64–67]. It is not known whether there is an inherited delayed maturation of disgust sensitivity in children with antisocial spectrum disorders. Adult psychopaths, however, do show downregulation of disgust sensitivity as they often participate in aberrant behaviors that expose them directly to various kinds of pathogenic infections.

If psychopaths vary in the degree to which they experience disgust relative to the general population, why is that? Here, we propose several testable hypotheses for future research on the biological correlates of psychopathy and suggest that deficits in disgust sensitivity may be a physiological marker of this particular personality disorder.

## 7 Hypotheses on psychopathy and disgust sensitivity

Extant data suggest that impulsivity (i.e., disinhibition of behavioral constraint) and sensation-seeking behavior are associated with psychopathy [68,69]. Impulsivity, in particular, may temporarily suppress disgust sensitivity to give in to stronger neurobiological drives, such as interpersonal violence and sexual desires. More specifically, we suggest that impulsive-irresponsible psychopathic traits may diminish or block the emotional manifestation of repulsion during the act of repetitive and persistent patterns of violent behavior. At the neural level, high impulsivity drive appears to be generated in the fear-alertness-aggression neurocircuitry of the amygdala, anterior insular cortex, and ventral-medial hypothalamus, respectively [70–72]. During emotional reactions, these three brain regions may interact to translate the emotions of disgust and impulsivity into particular outcomes. With recent technological advances [73], identifying the specific neural circuits between the amygdala, anterior insular cortex, and ventral-medial hypothalamus will help us to understand the different yet complementary brain mechanisms underlying both disgust and psychopathic behaviors.

**Figure 4:**
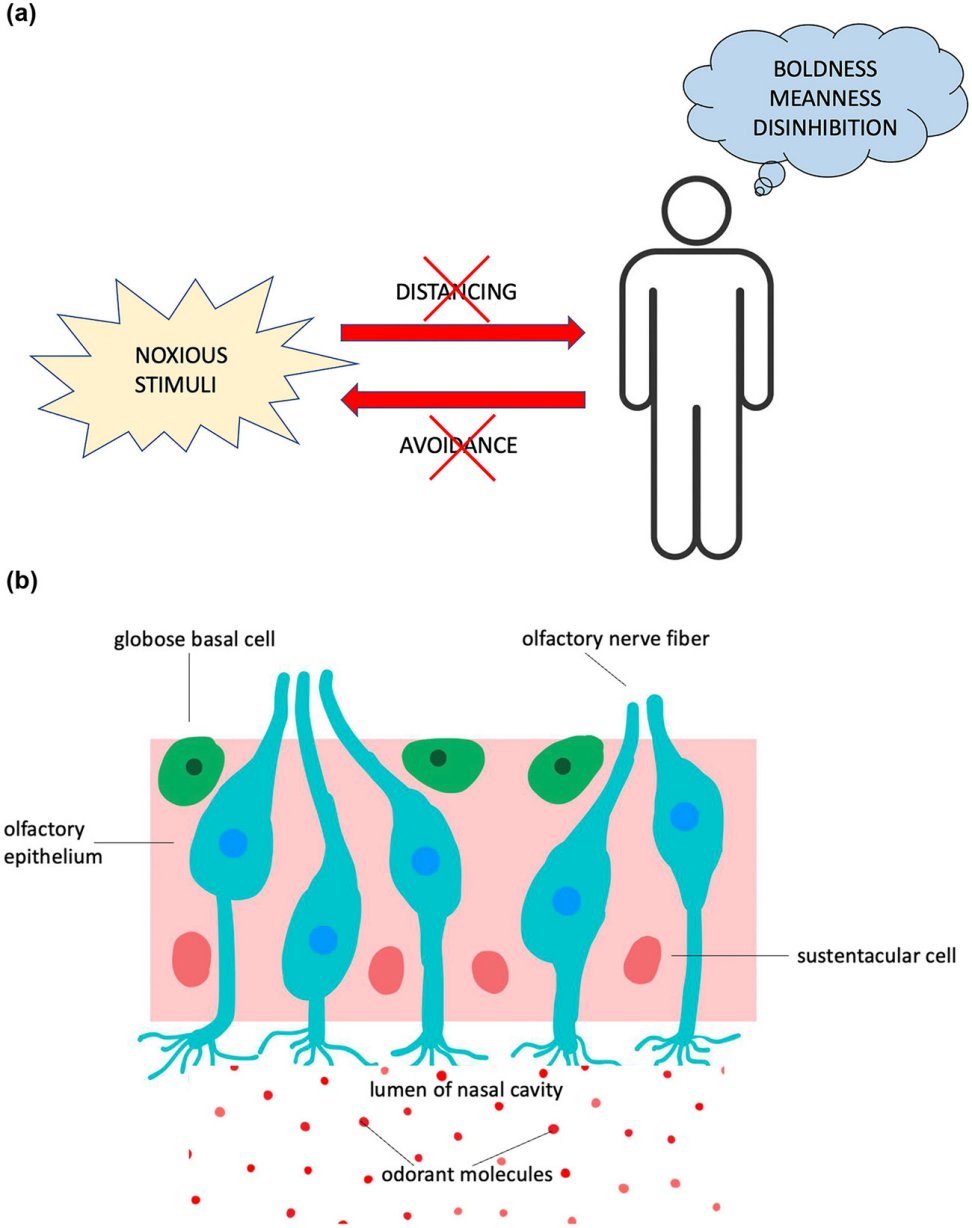
Psychopaths impulsively engage in repeated violent acts of criminality that expose them to a number of different pathogens and parasites. Thus, defense strategies or avoidance adaptations to limit infection in these individuals are either temporarily suppressed or permanently downregulated (*X*) to give in to stronger drives (a). This working hypothesis provides a new framework to advance the understanding of complex, multidimensional, and multifactorial human behaviors (e.g., meanness and disinhibition). We propose (among other causative factors) that fractures in olfactory function may contribute to psychopathy, as anosmia or contextual odor deficits have long been associated with neuropsychiatric disorders [74]. The olfactory epithelium is the only part of the CNS where nerve endings are directly exposed to the outside world (b). Additionally, olfactory neurons have direct access to neural circuits that control emotions (amygdala) and memories (hippocampus). Impaired discrimination of fouling odors (odorant molecules) within the brain may be a feature of psychopathy, as subjects with this personality disorder need to overcome functional barriers of disgust sensitivity to achieve rewarding experiences and exert self-control over their own awry emotions. Globose basal cell: neural progenitor in the olfactory epithelium. Sustentacular cell: this particular cell type provides the structural integrity of the olfactory epithelium. Olfactory neurons bundle together to form nerve fibers.

Sensory processing deficits, including olfactory deficits, may help explain the downregulation of disgust sensitivity observed in certain psychopaths. As described earlier, axillary odors emanating from steroids (e.g., androsterone), malodorous indoles from fecal material, and volatile organic compounds from cadaveric decomposition are unpleasant odorants that elicit not only disgust in most humans but also propel them to distance and avoid contact with putrid biological material or rotted corpses [2]. However, some psychopaths appear to be behaviorally immunized against such contextual background odorants (Figure 4a). As these subjects display impaired discrimination of odors, we suggest that there may be a deficit of odor perception akin to anosmia (Figure 4b). Anosmia, the inability to perceive odors (hyposmia), can result from traumatic brain injury, neurodegenerative diseases (e.g., Parkinson’s disease), or following a viral infection like COVID-19 (i.e., collectively referred to as acquired anosmia). In contrast, congenital anosmia can result from lesions of the olfactory epithelium or injury to mitral and tufted neurons, cells that project their myelinated axons to the piriform cortex, where the inherently subjective perception of smell is based [75,76]. Note that, although the experience of disgust is usually evoked by olfaction, the visual system is also included in the processing of disgust-relevant information. In this context, visual representation can be influenced by contextual odors, and olfaction signaling can be affected by visual cues [77,78]. Thus, there is a congruence between sensory systems, all of which may interact with variants in neurotransmitter pathway genes to activate subcortical (e.g., the amygdala) and cortical (e.g., orbitofrontal and anterior insula) brain networks involved in the emotional experience of disgust. Neuroimaging studies of odor perception could be used to delineate the disintegration of neural networks, which may underlie behaviors associated with psychopathy [79].

Although psychopathy is generally considered a behavioral disorder, any human behavior is the result of multiple biological networks, including possible contributions of the gastrointestinal tract to mood disorders, for example, and in the pathogenesis of certain psychiatric conditions. As discussed earlier, afferent fibers from the gastrointestinal tract and the vagus nerve coordinate sets of repulsive behaviors in response to poisonous food as well as contact with unseen pathogens. Based on this functional anatomical link, the importance of understanding the contribution of these afferent fibers to the development of a psychopathic trait is too important to ignore. Herein, we raise the possibility that deficits in the gastrointestinal tract and vagus nerve as a result of injury or inflammation upon exposure to certain microbes (also known as dysbiosis) can have an impact on the behavior of a given individual (e.g., disgust sensitivity) or on the indirect development of psychopathologies affecting the CNS. The vagus nerve is the main nerve of the parasympathetic division of the ANS. Sensory neurons of the vagus nerve detect a myriad of toxins that enter the gastrointestinal tract and transmit this emetic-based information to the medullary reticular formation of the hindbrain, leading to both nausea and vomiting [80,81]. Note that this emetic signal also extends to neural territories of the cerebral cortex (e.g., anterior insula) and limbic system (e.g., amygdala), where the emotional and cognitive processes of generalized fear and anxiety, oftentimes associated with the sensation of nausea, are registered. Thus, the process of nausea and vomiting involves the simultaneous signaling of the gut, including its enteric nervous system, the CNS, and the ANS. Cell signaling deficits may, therefore, take place along any of these anatomical circuits in the form of neural excitation or inhibition input, glial reactivity, oscillatory sensorimotor activity, or inflammatory injury. In this latter case, glucocorticoids (e.g., cortisol) secreted by the adrenal glands during stressful events affect neurons and glial cells in the enteric system to cause intestinal inflammation and bowel diseases [82]. Similarly, gut inflammation can result from the colonization of enteric pathogens, including proteobacteria such as *Salmonella* spp., *Shigella* spp., and *Yersinia* spp. [83–85]. It is assumed that enteric infections and human inflammatory diseases are characteristic traits of an abnormal microbiota caused either by a genetic predisposition or shaped by personal environmental variables such as diet and hygiene. Regardless, gut inflammation-associated dysbiosis has been implicated in disease progression and outcome, including certain psychiatric and neurological conditions [86,87]. The involvement of microbiota in gut disorders co-existing with psychopathologies should be considered as dysfunctions of the gastrointestinal tract may lead to perturbed disgust sensitivity in subjects with prolonged intestinal inflammation. The possibility that gut motility function, inflammatory events, and disgust sensitivity might be new features of psychopathy opens new avenues for further studies of the largest gathering of neurons and glia outside the CNS.

## 8 Conclusion

Disgust is a core human emotion evolved to detect and avoid poisonous food as well as contact with unseen pathogenic microorganisms. If a fracture in repulsive emotional flow is a feature of psychopathy, then dysregulation of this ancestral trait might also be identifiable in other psychopathologies as well [88,89]. Indeed, as illustrated in Figure 3b, *heightened* or *enhanced* levels of disgust are already noted in eating disorders, obsessive–compulsive disorders, certain specific phobias, posttraumatic stress disorders, and borderline personality disorders [90–93]. Thus, there is a precedence for disgust sensitivity being phenotypically related to other mental disorders, even if they do not share behavioral symptoms, disease comorbidities, or primary disease genes. The fact that there is an inverse relationship between psychopathy and the aforementioned mental disorders in terms of the overall level of disgust suggests that one may use such phenotypic dissimilarity to behaviorally predict and then test for the contribution of apparently unrelated genes to the same ancestral emotional trait. Obviously, we understand that psychopathy is not a unitary personality disorder, nor is disgust sensitivity the only personality trait seen in psychopaths. However, we strongly argue that deficits in disgust sensitivity could be an additional physiological marker of psychopathy, which requires further examination. New lines of research will be needed to clarify the exact role of each component of the disgust CNS-ANS-GUT anatomical circuit in psychopathy and determine whether the functional disgust abnormalities identified in psychopathy represent acquired signs of the personality disorder or vulnerable factors that increase the risk of developing antisocial spectrum disorders and use functional neuroimaging tools to survey cells that form part of a single interaction network and then assess genes that share one or more functional attributes related to brain and intestine activity. Finally, the hypotheses raised in this review explore a possible translational role of impaired disgust sensitivity in psychopathy with the aim to improve diagnosis or phenotypic recognition for a complex behavioral disorder with no specific treatment yet available.
